# Ontogeny of human IgE‐expressing B cells and plasma cells

**DOI:** 10.1111/all.12911

**Published:** 2016-06-08

**Authors:** F. Ramadani, H. Bowen, N. Upton, P. S. Hobson, Y.‐C. Chan, J.‐B. Chen, T. W. Chang, J. M. McDonnell, B. J. Sutton, D. J. Fear, H. J. Gould

**Affiliations:** ^1^ Randall Division of Cell and Molecular BiohphysicsKing's CollegeLondonUK; ^2^ Division of AsthmaAllergy and Lung BiologyKing's CollegeLondonUK; ^3^ Medical Research Council and Asthma UK Centre in Allergic Mechanisms in AsthmaLondonUK; ^4^ Genomics Research CenterAcademia SinicaTaipeiTaiwan

**Keywords:** allergy, germinal centre, human B cells, IgE class switching, plasma cell

## Abstract

**Background:**

IgE‐expressing (IgE^+^) plasma cells (PCs) provide a continuous source of allergen‐specific IgE that is central to allergic responses. The extreme sparsity of IgE^+^ cells *in vivo* has confined their study almost entirely to mouse models.

**Objective:**

To characterize the development pathway of human IgE^+^ PCs and to determine the ontogeny of human IgE^+^ PCs.

**Methods:**

To generate human IgE^+^ cells, we cultured tonsil B cells with IL‐4 and anti‐CD40. Using FACS and RT‐PCR, we examined the phenotype of generated IgE^+^ cells, the capacity of tonsil B‐cell subsets to generate IgE^+^ PCs and the class switching pathways involved.

**Results:**

We have identified three phenotypic stages of IgE^+^ PC development pathway, namely (i) IgE^+^germinal centre (GC)‐like B cells, (ii) IgE^+^ PC‐like ‘plasmablasts’ and (iii) IgE^+^ PCs. The same phenotypic stages were also observed for IgG1^+^ cells. Total tonsil B cells give rise to IgE^+^ PCs by direct and sequential switching, whereas the isolated GC B‐cell fraction, the main source of IgE^+^ PCs, generates IgE^+^ PCs by sequential switching. PC differentiation of IgE^+^ cells is accompanied by the down‐regulation of surface expression of the short form of membrane IgE (mIgE_S_), which is homologous to mouse mIgE, and the up‐regulation of the long form of mIgE (mIgE_L_), which is associated with an enhanced B‐cell survival and expressed in humans, but not in mice.

**Conclusion:**

Generation of IgE^+^ PCs from tonsil GC B cells occurs mainly via sequential switching from IgG. The mIgE_L_/mIgE_S_ ratio may be implicated in survival of IgE^+^ B cells during PC differentiation and allergic disease.

**Abbreviations**
BCR: B‐cell receptor
CSR: Class switch recombination
eGC: Early germinal centre
EMPD: Extramembrane proximal domain
FACS: Flow cytometry or fluorescence‐activated cell sorting
GC: Germinal centre
mIgE_L_: Long‐form membrane IgE
mIgE_S_: Short‐form membrane IgE
PB: Plasma blast
PC: Plasma cells
SHM: Somatic hypermutations

IgE antibodies mediate the activation of IgE effector cells and antigen‐presenting cells by allergen and hence are central to allergic disease 1, 2. The increasing prevalence of allergic disease is alarming, yet little is known about the mechanisms of IgE regulation. The sparsity of IgE^+^ B cells *in vivo* has hindered the attempts to investigate their development, particularly in the human system, while reliance on the results from mouse models often fails to predict the outcome of proposed therapies 3.

It is well established that T‐cell helper type 2 (Th2) cytokines, IL‐4 and/or IL‐13, in association with CD40 cross‐linking on B cells, promote class switch recombination (CSR) to IgE, which may be direct, from IgM to IgE, or sequential, via IgG 4. *In vivo,* CSR occurs in lymphoid tissues and at sites of inflammations 5, 6. In lymphoid tissue, B‐cell–T‐cell interactions lead to B‐cell proliferation and the formation of GCs, in which CSR is accompanied by somatic hypermutation (SHM) in the variable regions, culminating in affinity maturation and selection of the B cells of highest affinity for antigen*,* or allergen in the case of IgE 7, 8. The selected cells may recycle via the T‐cell compartment or differentiate into memory B cells and PCs to enter the circulation 9, 10.

Recent studies in the mouse revealed that the fate of IgE^+^ B cells is dramatically different from that of IgG1^+^ B cells, which express the most abundant and most thoroughly investigated isotype 11, 12, 13, 14, 15, 16. It was shown that although CSR to IgE is initiated in GCs, most of IgE^+^ cells exhibited a PC phenotype and were excluded from the GCs 14. Likewise, other studies of IgE in the mouse showed that IgE responses are more transient than those of IgG1 and were predominantly directed into the PC lineage 13. It was also reported that CSR pathway leading to IgE^+^ B cells determined their ultimate fate 16. Direct switching gave rise to IgE^+^ GC cells with an impaired B‐cell receptor (BCR) signalling, due to the low expression of the BCR, leading to cell death 16. This switching pathway was associated with the secretion of low‐affinity IgE antibodies 16, 17. In contrast, sequential switching generated IgE^+^ PCs with elevated BCR expression and was associated with the secretion of high‐affinity IgE antibodies 16, 17. It was inferred that the inheritance of SHM and affinity maturation from IgG1^+^ B cells are needed for the generation of a memory IgE response 16, 17.

The relevance of results in the mouse to human allergy has been questioned 18. For example, human IgE^+^ B cells express two forms, one short and one long form, of mIgE, mIgE_S_ and mIgE_L_ 19, 20. These mIgE isoforms arise from the alternative splicing of a common mRNA precursor, with mIgE_L_ containing a longer extra‐membrane proximal domain (EMPD) region, an additional 52‐amino acid residue between the C‐terminal Ig domain, Cε4 and the transmembrane M1 domain 19, 20, 21. Although nothing is yet known about the mechanisms that govern the relative expression of the two mIgE isoforms, there is evidence that the longer EMPD confers greater resistance to BCR‐induced apoptosis 21, 22.

We have previously characterized the capacity of various tonsil B‐cell subsets to undergo CSR to IgE *ex vivo* 23. Using this *ex vivo* tonsil human B‐cell culture system, we have now investigated the ontogeny of human IgE^+^ PCs. We point out many similarities, but also important differences from studies in the mouse models that may illuminate the mechanisms in allergy.

## Methods

### Isolation of human tonsil B cells

With informed written consent and ethical approval from Guy's Research Ethics Committee, we obtained human tonsils from donors undergoing routine tonsillectomies. Mononuclear cells were separated according to the density on a Ficoll gradient (GE Healthcare, Buckinghamshire, UK), and B cells were isolated using 2‐aminoethylisothiouronium bromide‐treated sheep red blood cells (TCS Biosciences Ltd, Buckingham, UK). B cells were >95% CD19^+^ as determined by flow cytometry analysis.

### Cell cultures

To induce CSR to IgE, B cells were cultured as previously described 23. Briefly, 0.5 × 10^6^ freshly isolated tonsil B cells were stimulated with IL‐4 (200 IU/ml unless otherwise stated; R&D Europe Systems Ltd, Abingdon, UK) and anti‐CD40 antibody (0.5 μg/ml unless otherwise stated; G28.5; American Type Culture Collection, Manassas, VA, USA) for up to 12 days.

### FACS analysis

Surface and intracellular staining of IgE^+^ cells was performed as previously described 23. A detailed account of FACS analysis, cell sorting, RNA isolation, qRT‐PCR and switch circle transcript PCR experiments can be found in the supplemental methods (available on the Allergy website).

### Statistical analysis

Statistical analysis was performed using the one‐way anova, with Bonferroni correction, unless otherwise stated. A value of *P* < 0.05 was considered significant (*P < 0.05, **P < 0.01, ***P < 0.001).

## Results

### Human IgE^+^ cells have three successive stages of differentiation into PCs

**Figure 1:**
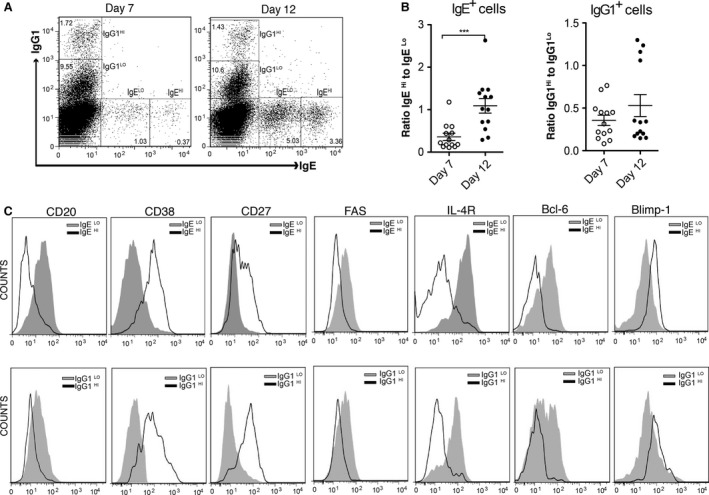
IgE^hi^ cells have a more differentiated phenotype than IgE^lo^ B cells. (A) Intracellular detection of two IgE^+^ and IgG1^+^ cell populations in our CSR to IgE cultures. Data are representative of five experiments. (B) The ratio of IgE^hi^ to IgE^lo^ and IgG1^hi^ to IgG1^lo^ cells was calculated using the frequency of these cells on days 7 and 12 of culture. Data represent the mean ± SD. ***P < 0.001 (two‐tailed *t*‐test). (C) Expression levels of CD20, CD27, CD38, Fas, IL‐4R, Bcl‐6 and Blimp‐1 on IgE^lo^ and IgG1^lo^ (filled histogram) *vs* IgE^hi^ and IgG1^hi^ (empty histograms) gated cells, respectively. Data are representative of six experiments.

To induce CSR to IgE, we cultured freshly isolated human tonsil B cells with IL‐4 and anti‐CD40 antibody. When staining for intracellular IgE, we consistently observe two IgE^+^ cell populations (Fig. 1A). We designate these IgE^lo^ and IgE^hi^ cells. Similarly, we observe two populations of IgG1^+^ cells, IgG1^lo^ and IgG1^hi^ cells. The ratio of IgE^hi^ to IgE^lo^ rose from day 7 to day 12 in culture, whereas that of IgG1^hi^ to IgG1^lo^ cells remained constant (Fig. 1A and B). On profiling the surface markers of these cells, by FACS, we observed that both IgE^hi^ and IgG1^hi^ express lower levels of CD20, Fas, IL‐4R and Bcl‐6 and higher levels of CD38, CD27 and Blimp‐1 than IgE^lo^ and IgG1^lo^ counterparts (Fig. 1C), indicating a more highly differentiated phenotype 24.

**Figure 2:**
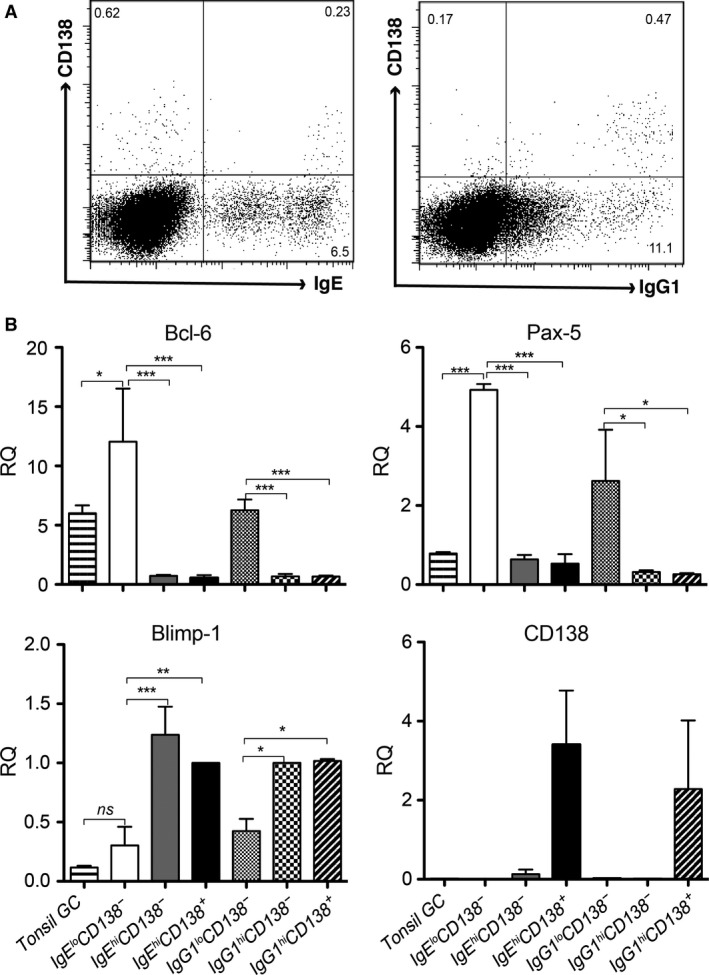
Human IgE^+^ and IgG1^+^ cells have three distinctive stages of differentiation into PCs. (A) On day 10 of total B cultures, the cells were surface‐stained for CD138 followed by fixation/permeabilization and intracellular staining for IgE and IgG1. The FACS dot plots show three distinct IgE^+^ and IgG1^+^ cell populations: IgE^lo^ CD138^−^, IgE^hi^ CD138^−^and IgE^hi^ CD138^+^ and the respective IgG1^+^ cell counterparts. (B) The three IgE^+^ and IgG1^+^ cell populations were FACS‐sorted into RNA processing buffer. The isolated RNA from these sorted cells was then used to determine the expression levels of the GC B‐cell markers, Pax5, Bcl6, and PC markers, Blimp1 and CD138, by qRT‐PCR. As controls, we used RNA cells of the FACS‐sorted tonsil GC B cells. Data represent the mean ± SEM and are derived from four different experiments. *P < 0.05, **P < 0.01, ***P < 0.001.

Two IgE^+^ cell populations were observed after the immunization of mice and were characterized as IgE^+^ GC cells and IgE^+^ PCs 13. However, unlike in mice, when staining the stimulated human B cells for CD138, a surface marker for the fully differentiated PCs, we observed three IgE^+^ cell populations: IgE^lo^CD138^−^, IgE^hi^ CD138^−^ and IgE^hi^CD138^+^ cells (Fig. 2A).

To characterize in more detail the IgE^+^ cell populations along their differentiation pathway into PCs, we used a fixation and permeabilization FACS procedure 25 to isolate RNA from sorted IgE^lo^CD138^−^, IgE^hi^CD138^−^ and IgE^hi^CD138^+^ cells. For comparison, we also sorted the IgG1^+^ cell counterparts. cDNA generated from these cells was used to quantify the relative expression levels of selected transcription factors known to be involved in maintaining the GC reaction and the B‐cell identity of the cells, or in inducing B‐cell differentiation towards PCs 7, 26. Figure 2B compares these expression levels with those on the unstimulated tonsil GC B‐cell controls.

Bcl‐6 and Pax‐5, important for maintaining the B‐cell identity and the GC phenotype 7, 26, 27, are abundantly expressed in the GC B cells and the IgE^lo^CD138^−^ and IgG1^lo^CD138^−^ cells, but strongly down‐regulated in both the IgE^hi^ (IgE^hi^CD138^−^ and IgE^hi^CD138^+^) and IgG1^hi^ (IgG1^hi^CD138^−^ and IgG1^hi^CD138^+^) cells (Fig. 2B). The opposite pattern is seen for Blimp‐1 expression, an important factor of PC differentiation and function 7, 26, 28. As the FACS‐derived phenotype of these cells predicts, expression of the PC marker, CD138, is seen only in IgE^hi^CD138^+^ and IgG1^hi^CD138^+^ cells (Fig. 2B). There were, moreover, no discernible phenotypic differences between IgE^+^ and IgG1^+^ cells at different stages of differentiation. In addition, we find that as IgE^+^ and IgG1^+^ cells differentiate, they down‐regulate Ki‐67 (Fig. S1), a marker of proliferation, and their cell cycle progression declines, with the majority of the IgE^+^ and IgG1^+^ PCs being at the quiescent G_0_ stage of the cell cycle (Fig. S2). In sum, these observations, consistent with those on the cell surface markers, indicate that IgE^lo^ (IgE^lo^CD138^−^) cells have a phenotype with GC B cell‐like characteristics, whereas the IgE^hi^ cells represent a later stage of differentiation into a PC‐like (IgE^hi^CD138^−^) ‘plasmablast’ phenotype, and only a small proportion appear to be fully differentiated PCs (IgE^hi^CD138^+^).

### Maintenance of GC B cells contributes to increased yields of IgE^+^ PCs

**Figure 3:**
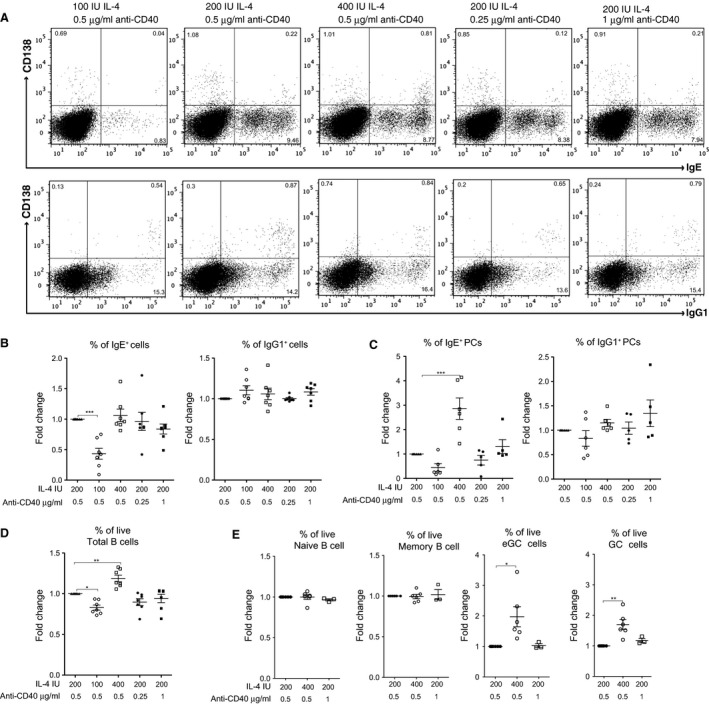
GC‐derived B cells and the yields of IgE^+^ PC are sensitive to changes in IL‐4 concentration. To determine the effect of IL‐4 and anti‐CD40 concentrations on the generation of IgE^+^ cells, especially IgE^+^ PCs, tonsil B cells were cultured with varying concentrations of IL‐4 and anti‐CD40. (A) On day 10 of culture, the cells were stained for IgE and CD138 (top panels) and IgG1 and CD138 (bottom panels). The percentages of total IgE^+^ and IgG1^+^ cells (B), IgE^+^ and IgG1^+^ PCs (C) and % of live cells in culture (D) were made relative to the 200 IU/ml of IL‐4 and 0.5 μg/ml of anti‐CD40 concentrations, standard concentrations used to induce CSR to IgE. (E) Percentage of live cells in cultures containing the FACS‐sorted naïve, memory, eGC and GC B cells cultured with increased concentrations of IL‐4 (400 IU/ml) or anti‐CD40 (1 μg/ml). The data show the fold changes in the percentage of live cells relative to the 200 IU/ml of IL‐4 and 0.5 μg/ml of anti‐CD40 cell culture condition and represent the mean ± SD. *P < 0.05, **P < 0.01, ***P < 0.001 (one‐way anova, Dunnett's test).

In IL‐4‐ and anti‐CD40‐stimulated tonsil B‐cell cultures, B cells from the GC compartments are the main sources of IgE^+^ cells 23. By comparison with naïve and memory B cells, these cells have very high rates of cell death, but also display an elevated expression of IL‐4R and CD40 23. We investigated the significance of these differences by stimulating human tonsil B cells with different IL‐4 and anti‐CD40 concentrations. Reducing the IL‐4 concentration from 200 IU/ml (the level customarily used to stimulate CSR to IgE) to 100 IU/ml reduced the percentage of IgE^+^ cells (Fig. 3A,B), revealing that CSR to IgE is sensitive to the IL‐4 concentration within this range of concentration. Increasing the concentration of IL‐4 from 200 to 400 IU/ml caused no further accretion of IgE^+^ cells, but engendered a threefold increase in the proportion of IgE^+^ PCs (Fig. 3A,B,C). In contrast, these changes in IL‐4 concentrations did not affect IgG1^+^ cells (Fig. 3A,B,C). Neither IgE^+^ nor IgG1^+^ cells evinced any response to changes in anti‐CD40 concentration (Fig. 3A,B,C). We also noted a significant reduction in the percentage of live cells cultured with 100 IU/ml of IL‐4, and a marked increase in cultures with 400 IU/ml of IL‐4 (Fig. 3D).

This observation led – because GC B cells are the main source of IgE^+^ cells 23 – to the conjecture that the action of IL‐4 may be restricted to the B cells from the GC compartments. To address this, we sorted tonsil B cells into naïve (CD27^−^CD38^−^CD77^−^), memory (CD27^+^CD38^−^CD77^−^), early GC (CD27^−^CD38^+^CD77^+^) and GC (CD27^+^CD38^+^CD77^+^) B cells 23 (Fig. S3) and cultured these cells with IL‐4 and anti‐CD40 (Fig. 3E). Again, unlike anti‐CD40, IL‐4 exerted a concentration‐dependent effect on GC‐derived B‐cell cultures, but not on naïve and memory B‐cell cultures (Fig. 3E). The percentage of live cells in GC B‐cell cultures rose when the concentration of IL‐4 was increased from 200 to 400 IU/ml (Fig. 3E). We conclude that IL‐4 contributes to the maintenance of the GC B cells and that the maintenance of these cells in culture results in higher yields of IgE^+^ PCs.

### Switched IgE^+^ cells from GC B‐cell cultures undergo rapid differentiation into IgE^+^ PCs

**Figure 4:**
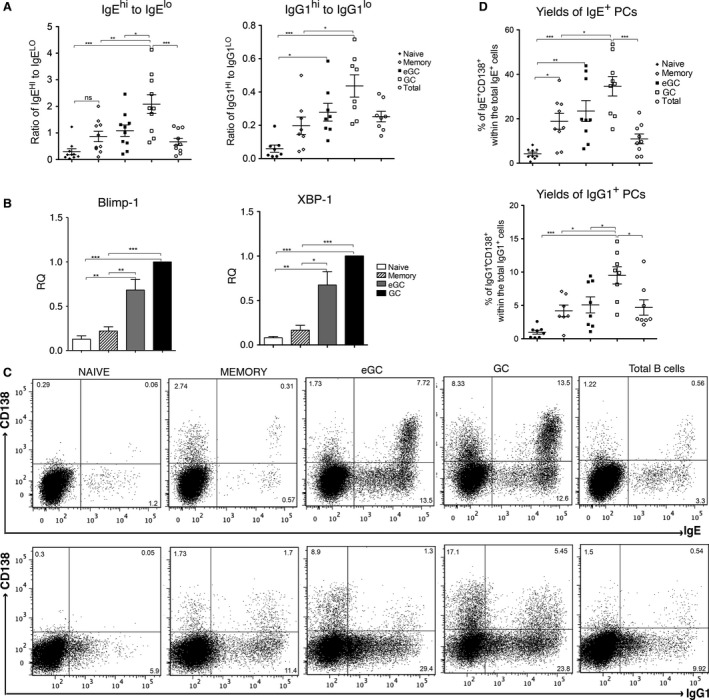
GC B‐cell cultures yield the highest percentage of IgE^+^ PCs. (A) Ratio of IgE^hi^ to IgE^lo^ cells and IgG1^hi^ to IgG1^lo^ cells in IL‐4‐ and anti‐CD40‐stimulated cultures of naïve, memory, eGC, GC and unfractionated total B cells. (B) Blimp‐1 and Xbp‐1 expression levels in sorted tonsil B‐cell subsets prior to culture with IL‐4 and anti‐CD40. Data represent the mean ± SD and are derived from three different experiments. (C) FACS dot plots show the CD138 expression on IgE^+^ cells (top panels) and IgG1^+^ cells (bottom panels) after 10 days of culture with IL‐4 and anti‐CD40. (D) Yields of IgE^+^ PCs (IgE^+^ CD138^+^) and IgG1^+^ PCs (IgG1^+^ CD138^+^) as a percentage of the total IgE^+^ and IgG1^+^ cells, respectively. *P < 0.05, **P < 0.01, ***P < 0.001.

The above results suggest that the GC environment may favour the generation of IgE^+^ PCs. To confirm this, we cultured tonsil naïve, memory, eGC and GC B cells with IL‐4 and anti‐CD40, with unfractionated total B cells as controls, and determined the IgE^hi^/IgE^lo^ cell ratio as a measure of the rate of differentiation of the newly switched human IgE^+^ cells. We found that this ratio was lowest in naïve (0.19 ± 0.14) and highest in GC B‐cell cultures (2.24 ± 1.1), whereas the memory (0.95 ± 0.59), eGC (1.18 ± 0.64) and total B‐cell cultures (0.67 ± 0.42) had IgE^hi^/IgE^lo^ cell ratios intermediate to that in the naïve and GC B‐cell cultures (Fig. 4A). IgG1^+^ cells, by contrast, despite their similar pattern of differentiation throughout the different B‐cell cultures (Fig. 4A), were predominantly IgG1^lo^ (0.06 ± 0.06–0.44 ± 0.19).

Examining the expression of Blimp‐1 and Xbp‐1, two important factors in PC differentiation and function 7, 26, 28, before culture, we found that eGC and GC B‐cell fractions expressed significantly higher levels of Blimp‐1 (Figs 4B and S4) and Xbp‐1 (Fig. 4B) than the other fractions. We hypothesized that the higher levels of Blimp‐1 and Xbp‐1 expression in eGC and GC B cells might predispose newly switched IgE^+^ cells, derived from these cells, towards the PC lineage. We therefore examined the PC differentiation of IgE^+^ cells in each of the sorted B‐cell cultures (Fig. 4C).

To account for the differences in CSR to IgE in these four different tonsil B‐cell fractions and the variability among the different tonsil B‐cell cultures 23, we measured the yields of IgE^+^ PCs as a fraction of all IgE^+^ cells in each culture (Fig. 4D). We found that the GC B‐cell culture yielded the highest proportion of IgE^+^ PCs and that naïve B‐cell cultures yielded the lowest (Fig. 4C,D). Yet, despite the higher expression of Blimp‐1 and Xbp‐1 in eGC compared to the memory cells, the two cell cultures yielded similar proportions of IgE^+^ PCs (Fig. 4C,D). The yields of IgE^+^ PCs in the unfractionated B‐cell cultures were between those in naïve and memory/eGC B‐cell cultures (Fig. 4C,D). These results show that elevated Blimp‐1 and Xbp‐1 expression cannot fully account for the rapid rate of PC differentiation.

The difference in the yields of IgG1^+^ PCs between the various B‐cell cultures showed a similar pattern to the yields of IgE^+^ PCs (Fig. 4C,D). However, despite this, we find that the yields of IgG1^+^ PCs were much lower than those of IgE^+^ PCs. This is also evident in naïve B‐cell cultures, which have no IgG1^+^ cells at the start of the culture 23, where the yields of IgE^+^ PCs are twice those of IgG1^+^ PCs (Fig. S5). The data demonstrate that IgE^+^ B cells have a much higher frequency of PC differentiation than IgG1^+^ cells.

### IgE^+^ PCs can be generated by both direct and sequential CSR

**Figure 5:**
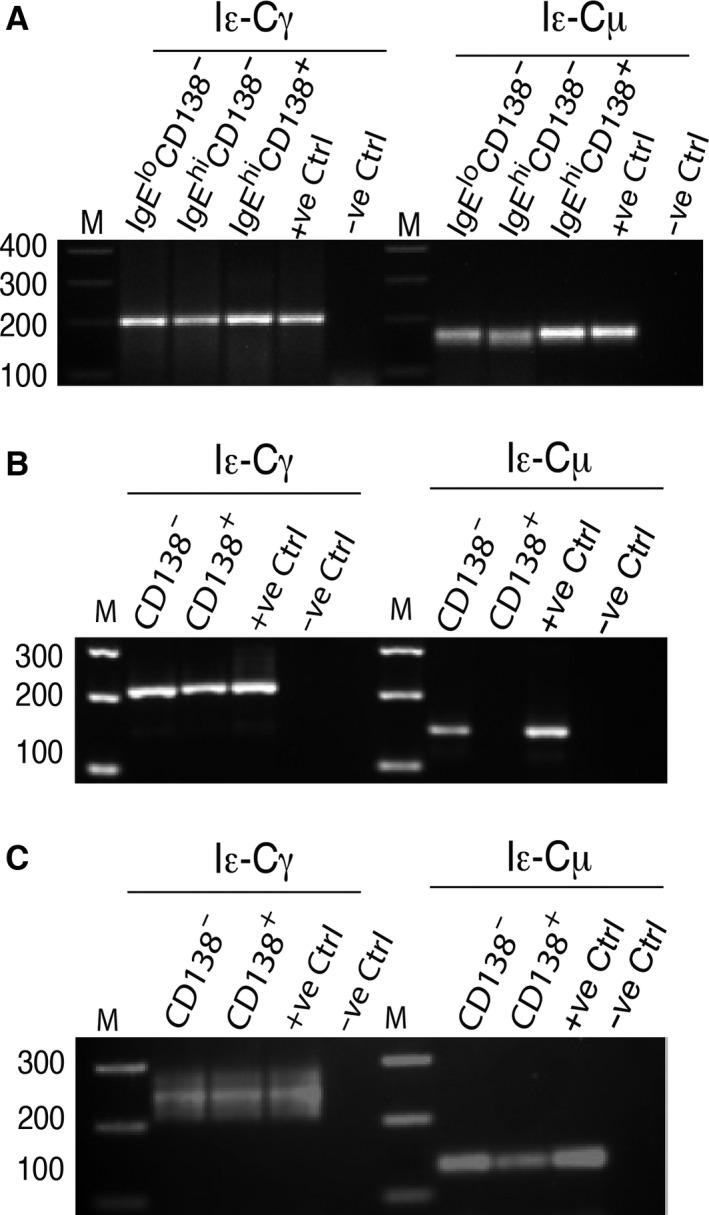
Both direct and sequential switching generate IgE^+^ GC B cells and IgE^+^ PCs. (A) RNA from the sorted IgE^lo^ CD138^−^, IgE^hi^ CD138^−^ and IgE^hi^ CD138^+^ was used for the detection of Iε‐Cμ SCT (167 bp; direct switching) and Iε‐Cγ SCT (202 bp; sequential switching) by nested PCR. The PCRs were standardized by using equal amounts of RNA for the cDNA synthesis. CD138^−^ and CD138^+^ cells were sorted from the IL‐4‐ and anti‐CD40‐stimulated cultures of enriched GC (CD38^+^) B cells (B) and naïve B‐cell cultures (C), and RNA was isolated and used for the analysis of IgE switching pathway as above. As a positive control, cDNA from an IL‐4‐ and anti‐CD40‐stimulated B‐cell culture that yielded high percentages of IgE^+^ cells was used, and as a negative control, dH_2_O. (M= marker). Data are representative of four different experiments.

Previous studies in the mouse demonstrated that sequential CSR to IgE from an IgG1^+^ B cell is required for the generation of high‐affinity IgE antibodies 17. A follow‐up report suggested that direct CSR from IgM to IgE generates IgE^+^ GC cells and sequential CSR from IgG to IgE leads to IgE^+^ PCs 16. To determine the relative importance of the CSR pathways in the generation of IgE^+^ cells in our *total* B‐cell cultures, we examined switch circle transcripts (SCTs) by a nested PCR. Analysis of SCTs shows that both IgM to IgE (Iε‐Cμ) and IgG to IgE (Iε‐Cγ) SCTs were present in IgE^lo^CD138^−^, IgE^hi^CD138^−^ and IgE^hi^CD138^+^ cells (Fig. 5A), revealing that both direct and sequential CSR can give rise to IgE^+^ GC B cells and IgE^+^ PCs.

Next, we investigated the CSR pathways that generate IgE^+^ PCs in the cultures of B cells from the GC and naïve compartments. We found that in GC B‐cell cultures, both Iε‐Cμ and Iε‐Cγ SCTs were present in CD138^−^ cells, but only Iε‐Cγ SCTs in the CD138^+^ cells (Fig. 5B). In contrast, in naïve B‐cell cultures, we detected both Iε‐Cμ and Iε‐Cγ SCTs in the CD138^−^ and CD138^+^ cells (Fig. 5C). Overall, these data demonstrate that in human tonsil B‐cell cultures, both direct and sequential CSR can give rise to IgE^+^ cells at all stages of differentiation. However, the generation of IgE^+^ PCs from the GC‐derived B cells appears to be selective, favouring sequential CSR to IgE. This may reflect the process of affinity maturation in IgG1^+^ GC B cells *in vivo*, leading to greater survival of cells expressing high‐affinity B cells, coordinated with CSR, and resulting in apoptosis of the less‐fit GC cells during *ex vivo* culture 9, 16, 17, 29, 30.

### Modulation of surface IgE expression along the differentiation pathway of IgE^+^ cells

**Figure 6:**
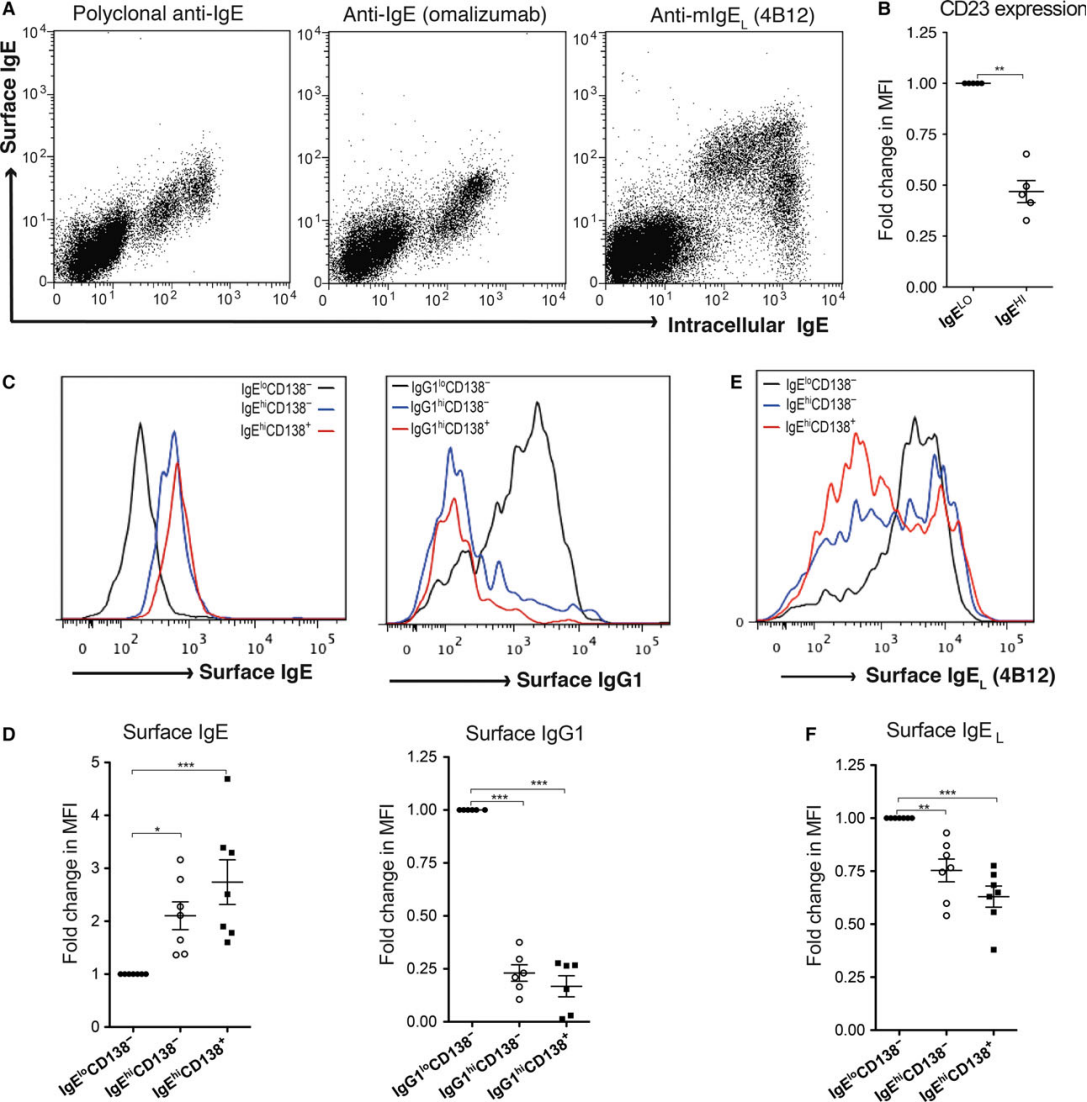
Surface mIgE expression along the differentiation pathway of IgE^+^ cells. (A) The FACS dot plot shows the day 12 cultured cells surface‐stained for IgE with a polyclonal anti‐IgE, anti‐IgE omalizumab and anti‐mIgE_L_ (4B12), followed by intracellular staining of IgE. Data are representative of five different experiments. (B) To determine the levels of membrane CD23 expression on IgE^lo^ and IgE^hi^ cells on day 12 of the culture, we surface‐stained for CD23, followed by fixation, permeabilization and intracellular staining of IgE. The expression level of CD23 is shown as the fold change in CD23 median fluorescence intensity (MFI), within the gated IgE^lo^ and IgE^hi^ cells, relative to the CD23 MFI on IgE^lo^ cells. (C) The histograms show the surface levels of mIgE and mIgG1 expression IgE^lo^ CD138^−^, IgE^hi^ CD138^−^, IgE^hi^ CD138^+^ and IgG1^lo^ CD138^−^, IgG1^hi^ CD138^−^, IgG1^hi^ CD138^+^, respectively. (D) Summarized surface expression levels of mIgE and mIgG1 at different stages of differentiation into PCs. Expression levels (MFI) were made relative to the levels on the IgE^lo^‐ and IgG1^lo^‐gated cells. (E) The histogram shows the surface expression levels of mIgE_L_ on IgE^lo^ CD138^−^ (black line), IgE^hi^ CD138^−^ (blue line) and IgE^hi^ CD138^+^ (red line). (F) Summarized surface expression levels of mIgE_L_ made relative to the expression levels on IgE^lo^ CD138^−^‐gated cells. Data represent the mean ± SD. *P < 0.05, **P < 0.01, ***P < 0.001 (one‐way anova, Dunnett's test).

In mouse IgG1^+^ GC B cells, surface IgG1 is expressed at a 20‐fold higher level than in IgG1^+^ PCs, whereas the opposite is true for the IgE^+^ cells 13, 16. When examining this phenomenon in human B cells, it is necessary to recall that there are two isoforms of human mIgE, mIgE_L_ and mIgE_S_, the latter being homologous to the one in the mouse. Stimulated human B cells express a preponderance of the mIgE_L_ isoform 19, 20, 21. To determine the surface expression of the two mIgE isoforms along the differentiation pathway of human IgE^+^ B cells, we used a combination of different anti‐IgE antibodies. Staining with either a polyclonal anti‐IgE, which recognizes all forms of IgE, or the anti‐IgE omalizumab, which recognizes only free IgE 31, revealed that surface mIgE expression was higher in IgE^hi^ than in IgE^lo^ cells (Fig. 6A). The similarity of staining by these two antibodies excludes the possibility that free exogenous IgE is binding to the low‐affinity IgE receptor, CD23, expressed on the B‐cell membrane, consistent with our observation that surface expression of membrane CD23 is lower on IgE^hi^ cells relative to IgE^lo^ cells (Fig. 6B).

Next, we determined the surface expressions of mIgE and mIgG1 at successive stages of IgE^+^ and IgG1^+^ cell differentiation, with the aid of, respectively, anti‐IgE omalizumab and a polyclonal anti‐IgG1 (Fig. 6C,D). It can be seen that surface mIgG1 is down‐regulated, and surface mIgE is up‐regulated in the course of PC differentiation in humans, as in mouse 13, 16. Importantly, however, staining with anti‐mIgE_L_, which recognizes only the mIgE_L_ 32, reveals a reduction in the surface mIgE on IgE^hi^ cells relative to that on IgE^lo^ cells (Fig. 6A). This result was confirmed by the concomitant reduction in surface mIgE_L_ along the differentiation pathway of IgE^+^ cells into PCs (Fig. 6E,F).

In sum, our data provide evidence that the down‐regulation of surface mIgE_L_ is compensated by the up‐regulation of surface mIgE_S,_ bearing in mind the elevated total surface mIgE expression.

## Discussion

Most of the recent insights into the biology of IgE have come from studies in the mouse 13, 14, 15, 16, 17, 33. The primary aim of our work was to elucidate the developmental pathway of IgE^+^ PCs in the human system. A secondary aim was to compare the results obtained using tonsil B cells with those reported recently from various mouse models.

The developmental pathway of human IgE^+^ cells can be resolved into three easily distinguishable stages, characterized as (i) IgE^+^ GC‐like (IgE^lo^CD138^−^), (ii) IgE^+^ PC‐like “plasmablasts” (IgE^hi^CD138^−^) and (iii) IgE^+^ PCs (IgE^hi^CD138^+^). The same developmental sequence was seen to prevail in the human tonsil IgG1^+^ cells. In contrast, mouse *in vivo* and *ex vivo* studies show the existence of only two distinct IgE^+^ (IgE^lo^CD138^−^ and IgE^hi^CD138^+^) and IgG1^+^ (IgG1^lo^CD138^−^ and IgG1^hi^CD138^+^) cell populations 13.

In the mouse, nascent IgE^+^ cells appeared to differentiate more swiftly into PCs than IgG1^+^ cells 13, 14, although this may have been their only route to survival 16, 33. Indeed, PC differentiation was the predominant fate of the mouse IgE^+^ cells *in vivo* as well as *ex vivo* 13, 14. Similarly, we show that in the human system, a greater proportion of IgE^+^ cells, compared with their IgG1^+^ counterparts, differentiate into PCs. The mechanisms contributing to the apparent propensity of IgE^+^ cells to differentiate into the PC lineage remain unclear.

The transgenic Blimp‐1‐deficient mouse B cells undergo CSR to IgE, but fail to differentiate into PCs 13, confirming that Blimp‐1 is not required for the CSR, but only for PC differentiation. However, Blimp‐1 expression in our IgE^+^ and IgG1^+^ cells is similar and therefore does not account for their differences in PC differentiation.

Both mIgG1 and mIgE have cytoplasmic tails that contribute to their enhanced signalling capacity 34, 35, 36, 37, 38. However, mIgE has a unique motif (YANIL‐motif) within its cytoplasmic tail, which is not found in the cytoplasmic tails of IgG isotypes, and binds proteins such as HS‐1 and HAX‐1 39. This could potentially explain the enhanced PC differentiation of IgE^+^ cells. We have previously shown that CD23 plays an important role in IgE synthesis 1. Therefore, the propensity of IgE^+^ cells towards the PC lineage could also result from the activity of CD23.

An important way in which the mouse and human systems diverge is that whereas mouse IgE^hi^ cells are mainly IgE^+^ PCs 13, 14, only a minor fraction of IgE^hi^ cells in total tonsil B cells become CD138^+^ PCs. It is possible that in these cultures, IgE^+^ PCs are generated, but fail to survive. However, we have demonstrated that increased IL‐4 concentration improved the yields of IgE^+^ PCs by maintaining the GC B cells. This implies that GC B cells are important for the generation of IgE^+^ PCs and that IL‐4 is essential not only for CSR to IgE but also for the maintenance of GC B cells, which have very high rates of cell death 23.

The high proportion of IgE^+^ cells that differentiated into IgE^+^ PCs at the end of the GC B‐cell cultures demonstrates the importance of GCs in the IgE^+^ PC generation. Yet, despite the different levels of Blimp‐1 and Xbp‐1 expression, eGC and memory B‐cell cultures display similar IgE^+^ cell propensity to PC differentiation, implying that other factors are important, for example the response to cytokines that affect the balance between cell division and cell death. In contrast, the majority of IgE^+^ switched cells in naïve B‐cell cultures, which undergo similar levels of CSR to IgE 23 and express similar levels of Blimp‐1 and Xbp‐1 to memory B cells, undergo a lower rate of PC differentiation. *In vivo* memory B cells are generated in GCs following SHM and affinity maturation, resulting in cells with high‐affinity BCRs 9, 10. The expression of a high‐affinity BCR is associated with the capacity for PC differentiation in the mouse 29, 30.

A novel observation in the mouse was the inheritance of IgE memory from IgG^+^ cells 13, 14, 16 and the different effects of direct and sequential switching on the fate of IgE^+^ cells 16, 17. The kinetics of B‐cell development revealed the relatively poor survival of IgE^+^ GC cells, attributed to their low level of mIgE expression, and impaired BCR signalling 13, 16. The conclusion was that IgE^+^ GC cells fail to undergo the canonical B‐cell differentiation programme that potentiates IgG1 memory immune responses 16. The fate of the mouse IgE^+^ cells was determined by their switching pathway; direct switching from IgM^+^ cells generated only a transient population of IgE^+^ GC cells, whereas switching from IgG1^+^ cells generated IgE^+^ PCs, accompanied by the up‐regulation of mIgE 16.

In contrast to the results in the mouse, we observe that *both* direct and sequential CSR can give rise to IgE^+^ GC B cells and IgE^+^ PCs in our tonsil B‐cell cultures. This is supported by studies on a chimeric mouse model containing the human M1′ sequence inserted into a murine ε gene reporter construct 12, 15. Their IgE^+^ GC B cells were longer‐lived than in other mouse models and able to differentiate into both IgE^+^ memory B cells and IgE^+^ PCs. Memory IgE^+^ B cells and IgE^+^ PCs can also be detected in the peripheral blood of humans 40. We and others have attributed this to the M1′ sequence, which protects against apoptosis 11, 12, 15, 18.

Furthermore, we have also presented evidence of only sequential CSR in IgE^+^ PCs generated from the human GC‐derived B‐cell cultures. The major implication of sequential CSR is the probability of affinity maturation of the IgG^+^ B‐cell precursors, by analogy to observations in the mouse 14, 16, 17. It may follow that sequential CSR is the predominant route to IgE in allergic disease 41. This is supported by the relative frequency of Iε‐Cγ and Iε‐Cμ transcripts in nasal biopsies from allergic rhinitis patients 42 and bronchial biopsies from asthma patients 43.

Our experiments also reveal the modulation of the surface mIgE_L_ and mIgE_S_ expression during PC differentiation. Earlier studies have shown that mIgE_L_ and mIgE_S_ are the predominant isoforms in the IL‐4‐ and anti‐CD40‐stimulated and unstimulated peripheral blood lymphocytes, respectively 19, 20, 21. Therefore, the expression levels of the two mIgE isoforms during the PC differentiation may reflect the levels of signalling and proliferation induced by the IL‐4 and anti‐CD40 stimulations. Nonetheless, the above considerations suggest that the regulation of mIgE_L_ expression is important for IgE homeostasis in the human system. Expression of this isoform is unique to humans and could well be one reason why allergic disease occurs naturally in humans, but apparently not in mice 18. These data are of particular interest because the EMPD of surface mIgE_L_ is a validated antibody target for immunotherapy of allergic disease, now under development in several laboratories with a view to therapeutic application 32, 44, 45.

In summary, using a tonsil *ex vivo* human system, we have investigated the ontogeny of IgE^+^ B cells and IgE^+^ PCs. We believe that our results have a direct relevance to the discovery of novel targets for the treatment of allergy.

## Author contributions

F.R. designed and performed experiments, analysed data and wrote the paper. H.B., N. U., P.S.H. and Y‐C.C. performed experiments and analysed data. J‐B.C. and T.W.C contributed vital new reagents and critically reviewed the manuscript. J.M.M. and B.J.S. provided guidance and critically reviewed the manuscript. D.J.F provided guidance, analysed data and critically reviewed the manuscript. H.J.G. designed experiments, analysed data and wrote the manuscript. All authors reviewed the final manuscript.

## Conflict of interest

The authors declare that they have no conflict of interests.

## Supporting information


**Data S1** Supplemental Methods

## References

[1] Gould HJ , Sutton BJ . IgE in allergy and asthma today. Nat Rev Immunol 2008;8:205–217. 18301424 10.1038/nri2273

[2] Dullaers M , De Bruyne R , Ramadani F , Gould HJ , Gevaert P , Lambrecht BN . The who, where, and when of IgE in allergic airway disease. J Allergy Clin Immunol 2012;129:635–645. 22168998 10.1016/j.jaci.2011.10.029

[3] Holmes AM , Solari R , Holgate ST . Animal models of asthma: value, limitations and opportunities for alternative approaches. Drug Discov Today 2011;16:659–670. 21723955 10.1016/j.drudis.2011.05.014

[4] Gould HJ , Beavil RL , Vercelli D . IgE isotype determination: epsilon‐germline gene transcription, DNA recombination and B‐cell differentiation. Br Med Bull 2000;56:908–924. 11359628 10.1258/0007142001903599

[5] Gevaert P , Nouri‐Aria KT , Wu H , Harper CE , Takhar P , Fear DJ et al. Local receptor revision and class switching to IgE in chronic rhinosinusitis with nasal polyps. Allergy 2013;68:55–63. 23157682 10.1111/all.12054

[6] Honjo T , Kinoshita K , Muramatsu M . Molecular mechanism of class switch recombination: linkage with somatic hypermutation. Annu Rev Immunol 2002;20:165–196. 11861601 10.1146/annurev.immunol.20.090501.112049

[7] Klein U , Dalla‐Favera R . Germinal centres: role in B‐cell physiology and malignancy. Nat Rev Immunol 2008;8:22–33. 18097447 10.1038/nri2217

[8] Vinuesa CG , Linterman MA , Goodnow CC , Randall KL . T cells and follicular dendritic cells in germinal center B‐cell formation and selection. Immunol Rev 2010;237:72–89. 20727030 10.1111/j.1600-065X.2010.00937.x

[9] Tarlinton DM , Smith KG . Dissecting affinity maturation: a model explaining selection of antibody‐forming cells and memory B cells in the germinal centre. Immunol Today 2000;21:436–441. 10953095 10.1016/s0167-5699(00)01687-x

[10] McHeyzer‐Williams LJ , McHeyzer‐Williams MG . Antigen‐specific memory B cell development. Annu Rev Immunol 2005;23:487–513. 15771579 10.1146/annurev.immunol.23.021704.115732

[11] Lafaille JJ , Xiong H , Curotto dLM . On the differentiation of mouse IgE(+) cells. Nat Immunol 2012;13:623. 22713817 10.1038/ni.2313

[12] Talay O , Yan D , Brightbill HD , Straney EE , Zhou M , Ladi E et al. Addendum: IgE+ memory B cells and plasma cells generated through a germinal‐center pathway. Nat Immunol 2013;14:1302–1304. 24240161 10.1038/ni.2770

[13] Yang Z , Sullivan BM , Allen CD . Fluorescent in vivo detection reveals that IgE(+) B cells are restrained by an intrinsic cell fate predisposition. Immunity 2012;36:857–872. 22406270 10.1016/j.immuni.2012.02.009

[14] Erazo A , Kutchukhidze N , Leung M , Christ AP , Urban JF Jr , Curotto de Lafaille MA et al. Unique maturation program of the IgE response in vivo. Immunity 2007;26:191–203. 17292640 10.1016/j.immuni.2006.12.006 PMC1892589

[15] Talay O , Yan D , Brightbill HD , Straney EE , Zhou M , Ladi E et al. IgE(+) memory B cells and plasma cells generated through a germinal‐center pathway. Nat Immunol 2012;13:396–404. 22366892 10.1038/ni.2256

[16] He JS , Meyer‐Hermann M , Xiangying D , Zuan LY , Jones LA , Ramakrishna L et al. The distinctive germinal center phase of IgE+ B lymphocytes limits their contribution to the classical memory response. J Exp Med 2013;210:2755–2771. 24218137 10.1084/jem.20131539 PMC3832920

[17] Xiong H , Dolpady J , Wabl M . Curotto de Lafaille MA, Lafaille JJ. Sequential class switching is required for the generation of high affinity IgE antibodies. J Exp Med 2012;209:353–364. 22249450 10.1084/jem.20111941 PMC3280879

[18] Gould HJ , Ramadani F . IgE responses in mouse and man and the persistence of IgE memory. Trends Immunol 2015;36:40–48. 25499855 10.1016/j.it.2014.11.002

[19] Peng C , Davis FM , Sun LK , Liou RS , Kim YW , Chang TW . A new isoform of human membrane‐bound IgE. J Immunol 1992;148:129–136. 1727861

[20] Zhang K , Saxon A , Max EE . Two unusual forms of human immunoglobulin E encoded by alternative RNA splicing of epsilon heavy chain membrane exons. J Exp Med 1992;176:233–243. 1613458 10.1084/jem.176.1.233 PMC2119292

[21] Batista FD , Efremov DG , Burrone OR . Characterization and expression of alternatively spliced IgE heavy chain transcripts produced by peripheral blood lymphocytes. J Immunol 1995;154:209–218. 7995941

[22] Poggianella M , Bestagno M , Burrone OR . The extracellular membrane‐proximal domain of human membrane IgE controls apoptotic signaling of the B cell receptor in the mature B cell line A20. J Immunol 2006;177:3597–3605. 16951319 10.4049/jimmunol.177.6.3597

[23] Ramadani F , Upton N , Hobson P , Chan YC , Mzinza D , Bowen H et al. Intrinsic properties of germinal center‐derived B cells promote their enhanced class switching to IgE. Allergy 2015;70:1269–1277. 26109279 10.1111/all.12679 PMC4744720

[24] Jourdan M , Caraux A , De Vos J , Fiol G , Larroque M , Cognot C et al. An in vitro model of differentiation of memory B cells into plasmablasts and plasma cells including detailed phenotypic and molecular characterization. Blood 2009;114:5173–5181. 19846886 10.1182/blood-2009-07-235960 PMC2834398

[25] Iglesias‐Ussel M , Marchionni L , Romerio F . Isolation of microarray‐quality RNA from primary human cells after intracellular immunostaining and fluorescence‐activated cell sorting. J Immunol Methods 2013;391:22–30. 23434645 10.1016/j.jim.2013.02.003 PMC3627819

[26] Martins G , Calame K . Regulation and functions of Blimp‐1 in T and B lymphocytes. Annu Rev Immunol 2008;26:133–169. 18370921 10.1146/annurev.immunol.26.021607.090241

[27] Horcher M , Souabni A , Busslinger M . Pax5/BSAP maintains the identity of B cells in late B lymphopoiesis. Immunity 2001;14:779–790. 11420047 10.1016/s1074-7613(01)00153-4

[28] Shaffer AL , Shapiro‐Shelef M , Iwakoshi NN , Lee AH , Qian SB , Zhao H et al. XBP1, downstream of Blimp‐1, expands the secretory apparatus and other organelles, and increases protein synthesis in plasma cell differentiation. Immunity 2004;21:81–93. 15345222 10.1016/j.immuni.2004.06.010

[29] Paus D , Phan TG , Chan TD , Gardam S , Basten A , Brink R . Antigen recognition strength regulates the choice between extrafollicular plasma cell and germinal center B cell differentiation. J Exp Med 2006;203:1081–1091. 16606676 10.1084/jem.20060087 PMC2118299

[30] Phan TG , Paus D , Chan TD , Turner ML , Nutt SL , Basten A et al. High affinity germinal center B cells are actively selected into the plasma cell compartment. J Exp Med 2006;203:2419–2424. 17030950 10.1084/jem.20061254 PMC2118125

[31] Shiung YY , Chiang CY , Chen JB , Wu PC , Hung AF , Lu DC et al. An anti‐IgE monoclonal antibody that binds to IgE on CD23 but not on high‐affinity IgE.Fc receptors. Immunobiology 2012;217:676–683. 22226669 10.1016/j.imbio.2011.11.006

[32] Chen JB , Wu PC , Hung AF , Chu CY , Tsai TF , Yu HM et al. Unique epitopes on C epsilon mX in IgE‐B cell receptors are potentially applicable for targeting IgE‐committed B cells. J Immunol 2010;184:1748–1756. 20083663 10.4049/jimmunol.0902437

[33] Laffleur B , Duchez S , Tarte K et al. Self‐Restrained B Cells Arise following Membrane IgE Expression. Cell Rep 2015;26:900–909. 10.1016/j.celrep.2015.01.023 25683713

[34] Engels N , Konig LM , Heemann C , Lutz J , Tsubata T , Griep S et al. Recruitment of the cytoplasmic adaptor Grb2 to surface IgG and IgE provides antigen receptor‐intrinsic costimulation to class‐switched B cells. Nat Immunol 2009;10:1018–1025. 19668218 10.1038/ni.1764

[35] Kaisho T , Schwenk F , Rajewsky K . The roles of gamma 1 heavy chain membrane expression and cytoplasmic tail in IgG1 responses. Science 1997;276:412–415. 9103199 10.1126/science.276.5311.412

[36] Achatz G , Lamers MC . In vivo analysis of the cytoplasmic domain of mIgE antibodies. Int Arch Allergy Immunol 1997;113:142–145. 9130505 10.1159/000237529

[37] Achatz G , Nitschke L , Lamers MC . Effect of transmembrane and cytoplasmic domains of IgE on the IgE response. Science 1997;276:409–411. 9103198 10.1126/science.276.5311.409

[38] Sato M , Adachi T , Tsubata T . Augmentation of signaling through BCR containing IgE but not that containing IgA due to lack of CD22‐mediated signal regulation. J Immunol 2007;178:2901–2907. 17312134 10.4049/jimmunol.178.5.2901

[39] Oberndorfer I , Schmid D , Geisberger R , Achatz‐Straussberger G , Crameri R , Lamers M et al. HS1‐associated protein X‐1 interacts with membrane‐bound IgE: impact on receptor‐mediated internalization. J Immunol 2006;177:1139–1145. 16818771 10.4049/jimmunol.177.2.1139

[40] Berkowska MA , Heeringa JJ , Hajdarbegovic E et al. Human IgE(+) B cells are derived from T cell‐dependent and T cell‐independent pathways. J Allergy Clin Immunol 2014;134:688–697. 24835500 10.1016/j.jaci.2014.03.036

[41] Looney TJ , Lee JY , Roskin KM , Hoh RA , King J , Glanville J et al. Human B‐cell isotype switching origins of IgE. J Allergy Clin Immunol 2015;137:579–586. 26309181 10.1016/j.jaci.2015.07.014 PMC4747810

[42] Takhar P , Smurthwaite L , Coker HA , Fear DJ , Banfield GK , Carr VA et al. Allergen drives class switching to IgE in the nasal mucosa in allergic rhinitis. J Immunol 2005;174:5024–5032. 15814733 10.4049/jimmunol.174.8.5024

[43] Takhar P , Corrigan CJ , Smurthwaite L , O'Connor BJ , Durham SR , Lee TH et al. Class switch recombination to IgE in the bronchial mucosa of atopic and nonatopic patients with asthma. J Allergy Clin Immunol 2007;119:213–218. 17208604 10.1016/j.jaci.2006.09.045

[44] Brightbill HD , Jeet S , Lin Z , Yan D , Zhou M , Tan M et al. Antibodies specific for a segment of human membrane IgE deplete IgE‐producing B cells in humanized mice. J Clin Invest 2010;120:2218–2229. 20458139 10.1172/JCI40141 PMC2877936

[45] Feichtner S , Infuhr D , Achatz‐Straussberger G , Schmid D , Karnowski A , Lamers M et al. Targeting the extracellular membrane‐proximal domain of membrane‐bound IgE by passive immunization blocks IgE synthesis in vivo. J Immunol 2008;180:5499–5505. 18390733 10.4049/jimmunol.180.8.5499 PMC2959155

