# Supplementary material for: Ontogeny of human IgE‐expressing B cells and plasma cells
Source: Allergy. 2016 Jun 8;72(1):66–76. doi: 10.1111/all.12911 (PMC5107308; doi:10.1111/all.12911)
Supplement: Supplementary file 1 — Data S1 Supplemental Methods [file ALL-72-66-s001.doc]

**Supplemental Materials**

**Ontogeny of Human IgE-expressing B Cells and Plasma Cells**

Faruk Ramadani *^,†,‡^,, Holly Bowen *^,†,‡^, Nadine Upton *^,†,‡^, Philip S Hobson *^,‡^, Yih-Chih Chan *^,†,‡^, Jiun-Bo Chen ^††^, Tse Wen Chang ^††^, James M McDonnell*^,†,‡^, Brian J Sutton*^,†,‡^, David J Fear *^,‡^, and Hannah J Gould*^,†,‡^

**Supplemental Methods**

**FACS analysis**

The following Abs were from Biolegend: anti-CD19 Alexa-647 (HIB19), anti-CD23 APC (EBVCS-5), anti-IgM APC (MHM880), anti-CD138 Brilliant violet 711, whereas anti-IgG1 PE/APC, anti-CD38 PE/Biotin and anti-CD138 APC were from Miltenyi Biotech. We also used anti-Bcl-6 (BCL-UP) APC and anti-Blimp-1 APC from eBiosceinces and R&D, respectively. The FITC anti-IgE polyclonal ab was from Vector Laboratories, the APC anti-IgE (Ige21) was from eBiosceinces, whereas the anti-IgE omalizumab (Novartis, Xolair), a humanized anti-IgE antibody, was conjugated with alexa-488 kit as per manufactures instructions (ALEXA 488 5-SDP Ester, Invitrogen). The anti-mIgE_L_ (4B12) biotin was produced in house[^1^](#_ENREF_1) (Institute of Molecular Medicine, National Tsing Hua University, Taiwan). Surface and intracellular staining of IgE^+^ cells was done as previously described[^2^](#_ENREF_2). For intracellular staining, cells were fixed with 2% paraformaldehyde (Electron Microscopy Sciences) for 10min at 37°C, washed, and re-suspended in permeabilization buffer (PBS, 0.5% Triton X-100, and 0.5% saponin (Sigma-Aldrich Ltd) for 20 min at RT. The cells were then washed and stained with fluorescently conjugated antibodies in the dark for 30 min. To determine the cell viability of the cultured cells we used the live/dead fixable dead stain kit (Life Technologies Ltd). Data was collected on a BD FACSCanto™ (BD Biosciences) and events were analysed using FlowJo software version 7.6.3 (Tree Star). To determine the cell cycle distribution and the quiescence status of IgE^+^ and IgG1^+^ cells we first stained the cells with anti-CD138 PE/Cy7 (Biolegend). The cells were then fixed and permeabilised as above, washed with PBS and treated with RNAse A (Sigma Aldrich Ltd) at 37C for 15 min followed by staining with anti-Ki67 FITC (BD Biosciences), 4',6-diamidino-2-phenylindole (DAPI), anti-IgG1 PE and anti-IgE APC for 30 mins at RT.

**Cell sorting**

*FACS sorting of tonsil B cells for culturing:* Following isolation, human tonsil B cells were surface stained for CD27 and CD38 on ice for 15-30 min. B cells were then sorted according to their expression of these surface molecules into naïve (CD27^-^CD38^-^), eGC (CD27^-^CD38^+^), GC (CD27^+^CD38^+/++^) and memory (CD27^+^CD38^-^) B cells using a BD FACSAria machine (BD Biosciences). Cells were collected into sterile FACS tubes containing FCS supplemented with 100 IU/mL penicillin, 100μg/mL streptomycin, 2mM glutamine (Invitrogen). Sorted cells were counted, washed and cultured with IL-4 (200 IU/mL; R&D Europe Systems Ltd) and anti-CD40 antibody (0.5 μg/mL; G28.5; American Type Culture Collection) or as indicated.

*FACS sorting of cultured cells for RNA isolation:*  To isolate the RNA from fixed and permeabilised cells we used methods similar to those that were previously reported [^3^](#_ENREF_3). In brief, on day 10 of the cell culture, the cells were harvested and the dead cells were removed by density on a Ficoll gradient (GE Healthcare). The cell suspension was then stained with a live/dead fixable stain dye (Life Technologies Ltd, Paisley, United Kingdom) and anti-CD138 conjugated antibodies followed by fixation with 2% paraformaldehyde. Cells were then washed twice with RNAsecure (Life Technologies Ltd) treated PBS, supplemented with 1% molecular grade BSA (Life Technologies Ltd), and permeabilised, for 30 mins on ice, with 1% molecular grade triton x100 (Sigma-Aldrich Ltd) containing 250 U/mL of RiboSafe RNase inhibitor (Bioline Reagents Ltd, London, United Kingdom) and 5 mM DL-dithiothreitol (Sigma-Aldrich Ltd). Following one wash with RNAsecure treated PBS, containing 100 U/mL of RNase inhibitor and 5 mM DL-dithiothreitol, cells were intracellularly stained for IgE and IgG1 for 45 min on ice. The IgE^lo^CD138^-^, IgE^hi^CD138^-^ and IgE^hi^CD138^+^cells and their respective IgG1 counterparts were FACS sorted into melting buffer (Invitrogen) containing 1600 U/mL RiboSafe RNase inhibitors and 10 mM DL-dithiothreitol and used for total RNA extraction (see below). In addition, to determine the class switching pathway giving rise to IgE^+^ PCs in the GC B cell cultures, after the presence of IgE^+^ PCs was confirmed, cells from these cultures were harvested and then stained with DAPI and anti-CD138 conjugated antibody. The DAPI^-^CD138^-^ and DAPI^-^CD138^+^ cell populations were FACS sorted into tubes containing RTL buffer (Qiagen, Crawley, UK) and used for total RNA extraction.

**Total RNA isolation and cDNA synthesis**

To isolate total RNA was from fixed and permeabilised cultured human tonsil B cells we used a modified protocol for the PureLink FFPE total RNA isolation kit (Invitrogen). Briefly, cells were sorted into the melting buffer containing 1600 U/mL RNase inhibitor (Bioline) and 10 mM DTT (Sigma-Aldrich Ltd), samples were stored at -80^0^C before proceeding to the proteinase K treatment for 15 min at 60^0^C. Subsequently the manufacturers instructions were followed, including the optional DNase digestion. The RNA was further cleaned up using the RNeasy Mini Kit RNA Cleanup protocol (Qiagen, Crawley, United Kingdom). RNA concentrations were measured using the NanoDrop 2000 (Thermo Scientific) and RNA integrity assessed using the 2100 Bioanalyser instrument (Agilent Technologies, Inc). To isolate total RNA from non-fixed/non-permeabilised cells we used the Qiagen RNeasy mini kit with on-column DNase digestion (Qiagen). The RNA was reverse transcribed with the Maxima® Reverse Transcriptase enzyme (Fermentas GmbH).

**Quantitative reverse transcriptase PCR (qRT-PCR)**

qRT-PCR was performed on Viia7 Real-Time PCR system (Life Technologies) according to manufacturers instructions. β−2-microglobulin (Applied Biosystems, Life Technologies Corporation) was used as an endogenous control. Gene specific primer/probe sets were designed using the on-line Universal ProbeLibrary Assay Design Centre (Roche Applied Science, Burgess Hill, UK). Universal ProbeLibrary probes were purchased from Roche Applied Science and corresponding forward and reverse primers synthesized by Sigma (Sigma-Aldrich Company Ltd, Dorset, UK). The primer/probe sets used were as follows; Bcl-6 (Fw- 5’-TTCCGCTACAAGGGCAAC-3’, Rev-5’-TGCAACGATAGGGTTTCTCA-3’, UPL 5), Pax-5 (Fw-5’-GA CGCTGACAGG GATGGT-3’, Rev-5’-CTCCAGGAGTCGTTGTACGAG-3’, UPL 83), Blimp-1 (Fw-5’-ACGTGTG GGTACGACCTTG-3’, Rev-5’-CTGCCAATCCCTGAAACCT-3’, UPL 67), CD138 (Fw-5’-AGGATGGAGGTCCTTCTGC-3’, Rev-5’-CCGAGGTTTCAAAGGTGAAGT-3’, UPL 66). Expression Suite Software v1.0.3 (Life Technologies) was used to determine relative quantification of the target cDNA according to the comparative C_T_ (ΔΔC_T_) method.

**Detection of I*ε-*Cμ and I*ε-*Cγ switch circle transcripts (SCTs)**

To determine the class switching pathways involved in giving rise to our IgE^+^ cells we examined the presence of SCTs, spanning from *Iε* to *IgHM* (I*ε-*Cμ; SCT for direct switching) or to *IgHG* (I*ε-*Cγ; SCT for sequential switching) using a nested PCR as previously described [^2^](#_ENREF_2). SCTs were amplified from cDNA using two rounds of nested PCR reaction: PCR1 and PCR2. In PCR1, 20 μl of reaction mix contained 1 μl cDNA, 250nM of IεF1 (5’-CCACGGTTACTGATCATCTGGGAGC-3’) and 250nM of CμR1 (5’-CGTTGCTGAGGGAGTAGAGTCC-3’) for Iε-Cμ CT or CγR1 (5’-CCACGCTGCTCGTATCCGAC-3’) for Iε-Cγ CT in 1x [Phusion Flash High-Fidelity PCR Master Mix](http://www.thermoscientificbio.com/pcr-enzymes-master-mixes-and-reagents/phusion-flash-high-fidelity-pcr-master-mix/) (Thermo Scientific, UK). PCR1 reactions were performed at 98^o^C for 10 sec, followed by 30 cycles of 98^o^C for 1 sec; 68^o^C (Iε-Cγ) or 65^o^C (Iε-Cμ) for 10 sec; 72^o^C for 15 sec, and 1 cycle at 72^o^C for 1 min. In PCR2, 1μl of PCR 1 product was re-amplified in a 20μl reaction mix, using 250nM of IεF2 (5’-CTGAT CATCTGGGAGCTGTCC-3’) in conjunction with 250nM of CμR2 (5’-GGGGA ATTCTCACAGGAGAC-3’) for Iε-Cμ or CγR2 (5’-CACCGTCAC CGGTTCGGGG-3’) for Iε-Cμ in 1x [Phusion Flash High-Fidelity PCR Master Mix](http://www.thermoscientificbio.com/pcr-enzymes-master-mixes-and-reagents/phusion-flash-high-fidelity-pcr-master-mix/). PCR2 reaction was carried out at 98^o^C for 10 sec, followed by 20 cycles of 98^o^C for 1 sec; 68^o^C (Iε-Cγ) or 65^o^C (Iε-Cμ) for 5 sec; 72^o^C for 15 sec, and 1 cycle at 72^o^C for 1 min. PCR reactions were standardized by using equal amounts of RNA for the cDNA synthesis. GAPDH was amplified to check the integrity of cDNA.

**Supplemental Figures**

**
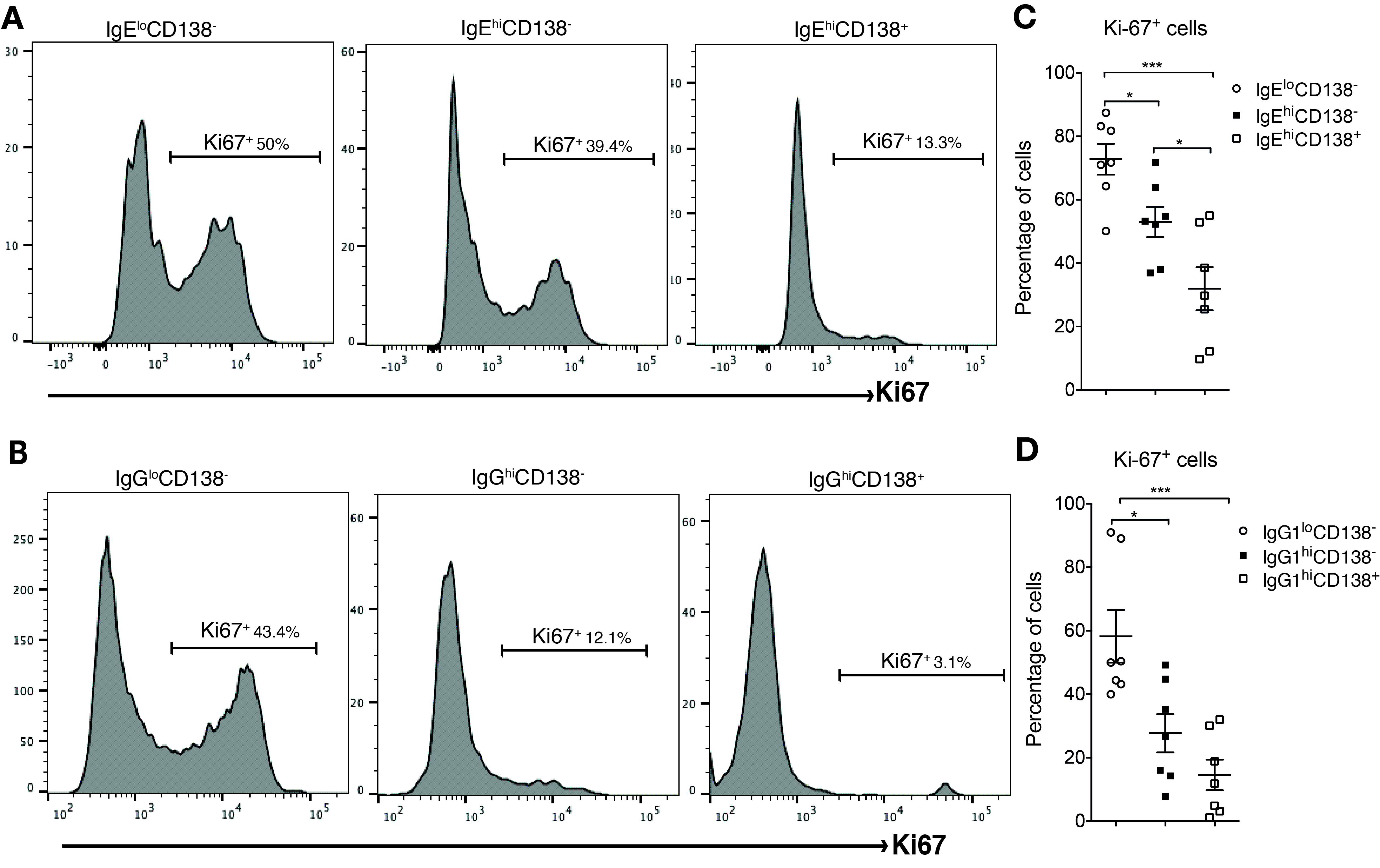
**

**Figure S1.** **Ki67 expression along the IgE^+^ and IgG1^+^ cell differentiation pathway into PCs.** (**A**) Histograms show the percentage of Ki67^+^ on IgE^+^ (IgE^lo^CD138^-^, IgE^hi^CD138^-^, IgE^hi^CD138^+^) and (**B**) IgG1^+^ cells (IgG1^lo^CD138^-^, IgG1^hi^CD138^-^, IgG1^hi^CD138^+^). The data are derived from day 10 of total B cell culture and summarized in (**C** and **D**).


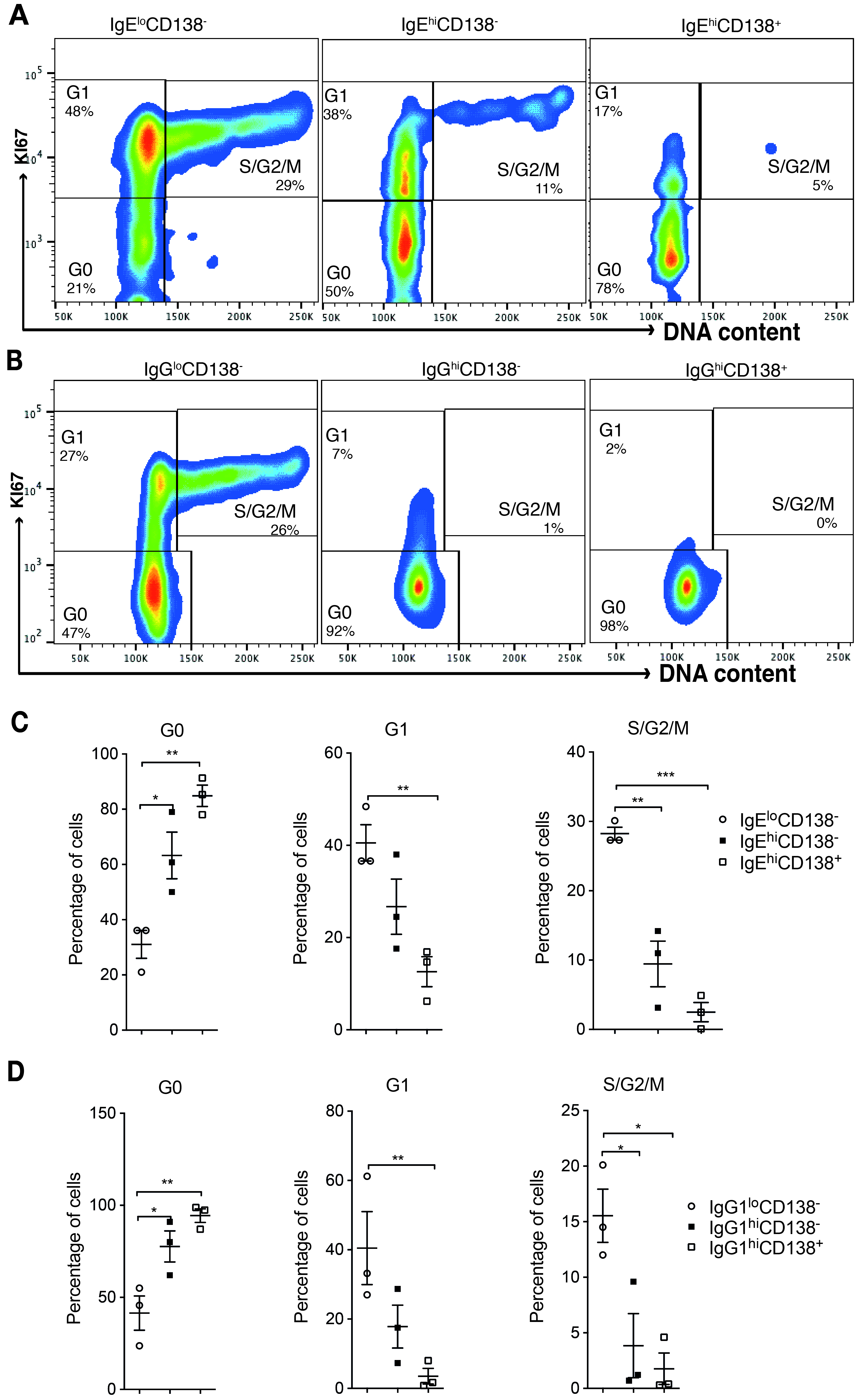


**Figure S2. Cell cycle distribution and quiescence status of the IgE^+^ and IgG1^+^ cells along their differentiation pathway into PCs.** Flow cytometry analysis of cell cycle analysis was performed by staining the DNA content with DAPI. In addition, we also measured the Ki67 expression in order to distinguish the dividing cells (G_1_ and S+G_2_/M) from quiescent (G_0_) cells. Cell cycle distribution and quiescence status of IgE^+^ cells (IgE^lo^CD138^-^, IgE^hi^CD138^-^, and IgE^hi^CD138^+^) is shown in (**A**), whereas (**B**) shows that of IgG1^+^ cells (IgG1^lo^CD138^-^, IgG1^hi^CD138^-^, and IgG1^hi^CD138^+^). The summary of data from three different experiments is shown in (**C** and **D**).


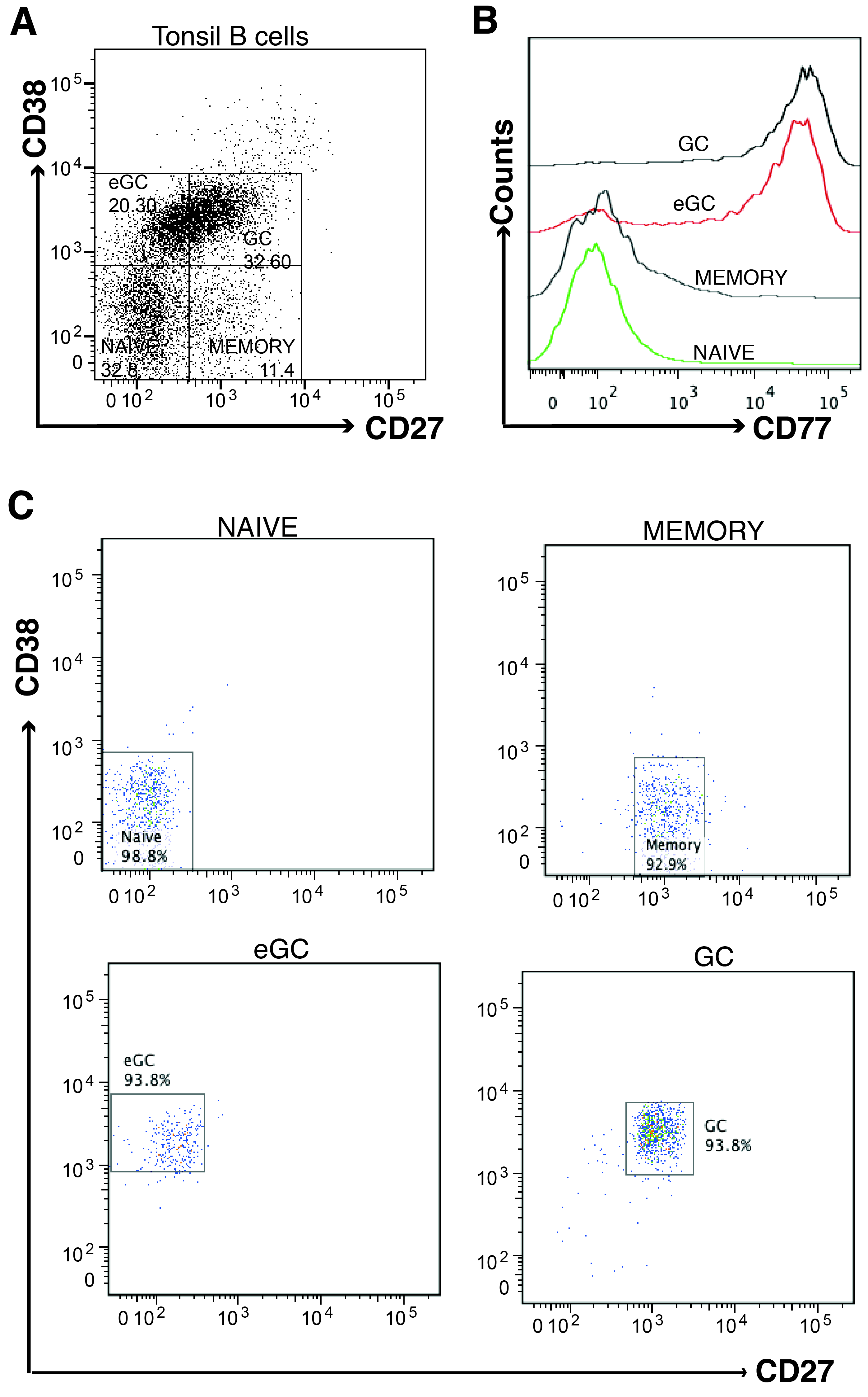


**Figure S3.** **FACS sorting of tonsil B cell fractions. (A)** CD19^+^ tonsil B cells comprises a mixture of B cell subsets which can be distinguished by their surface levels of CD27 and CD38 expression. (**B**) In contrast to naïve (CD27^-^CD38^-^) and memory B cells (CD27^+^CD38^-^), B cells from the GC of the tonsils express high levels of CD77. (**C**) Tonsil B cells were FACS sorted based on their CD27 and CD38 surface expression into naïve (CD27^-^CD38^-^), eGC (CD27^-^CD38^+^), GC (CD27^+^CD38^+/++^) and memory B cells (CD27^+^CD38^-^). The sorted cells were then cultured for up to 10 days with IL-4 and anti-CD40.


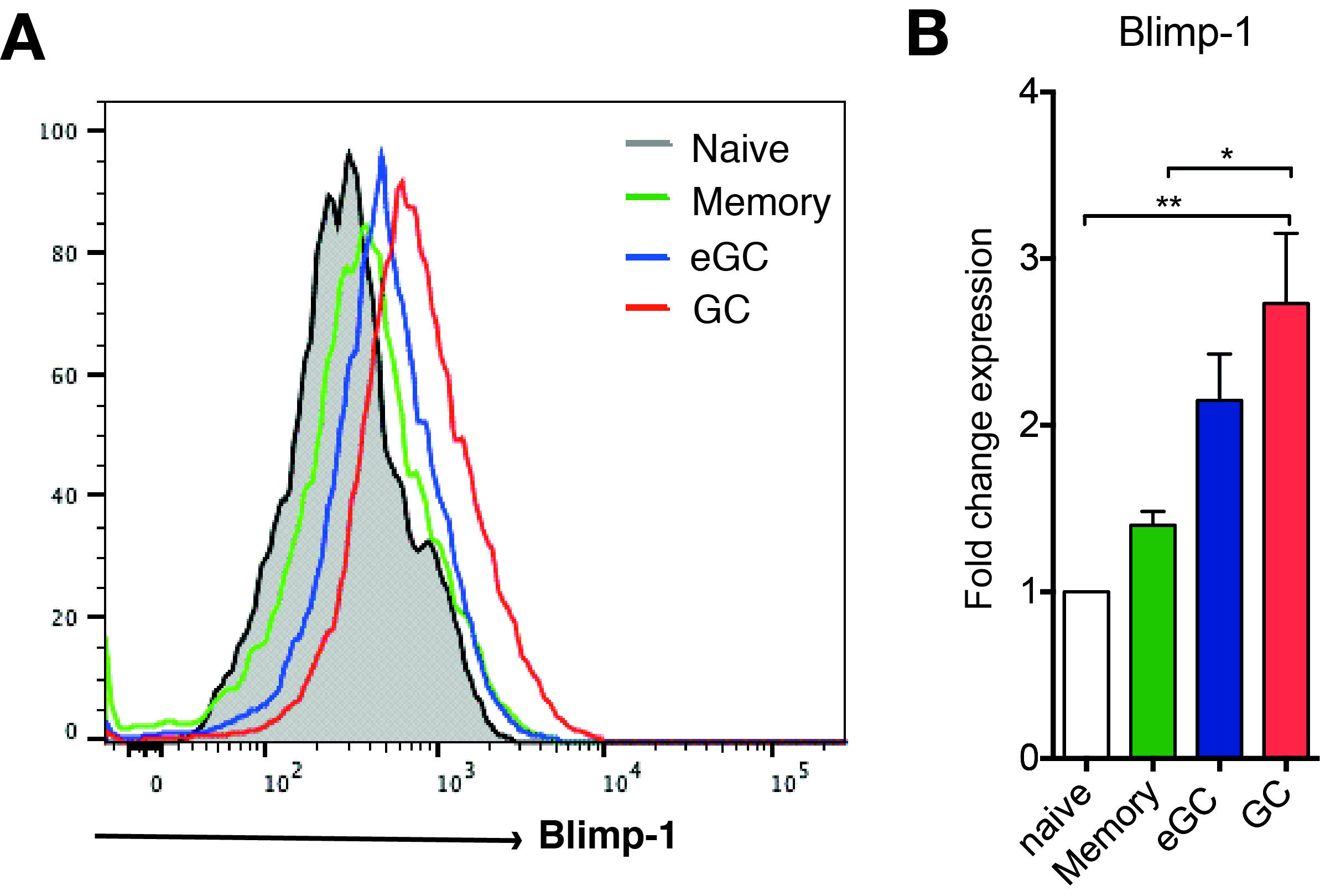


**Figure S4.** **Blimp-1 expression in tonsil B cell subsets.** To determine the levels of Blimp-1 expression, we surface stained tonsil B cells with anti-CD27 and anti-CD38 followed by intracellular staining with anti-Blimp-1. (**A**) Histograms show the levels of Blimp-1 expression in different tonsil B cell subsets prior to culture with IL-4 and anti-CD40. (**B**) Data show the fold change of anti-Blimp-1 stained cells relative to naïve B cells. They represent the mean +/- SD and are derived from 3 different experiments.


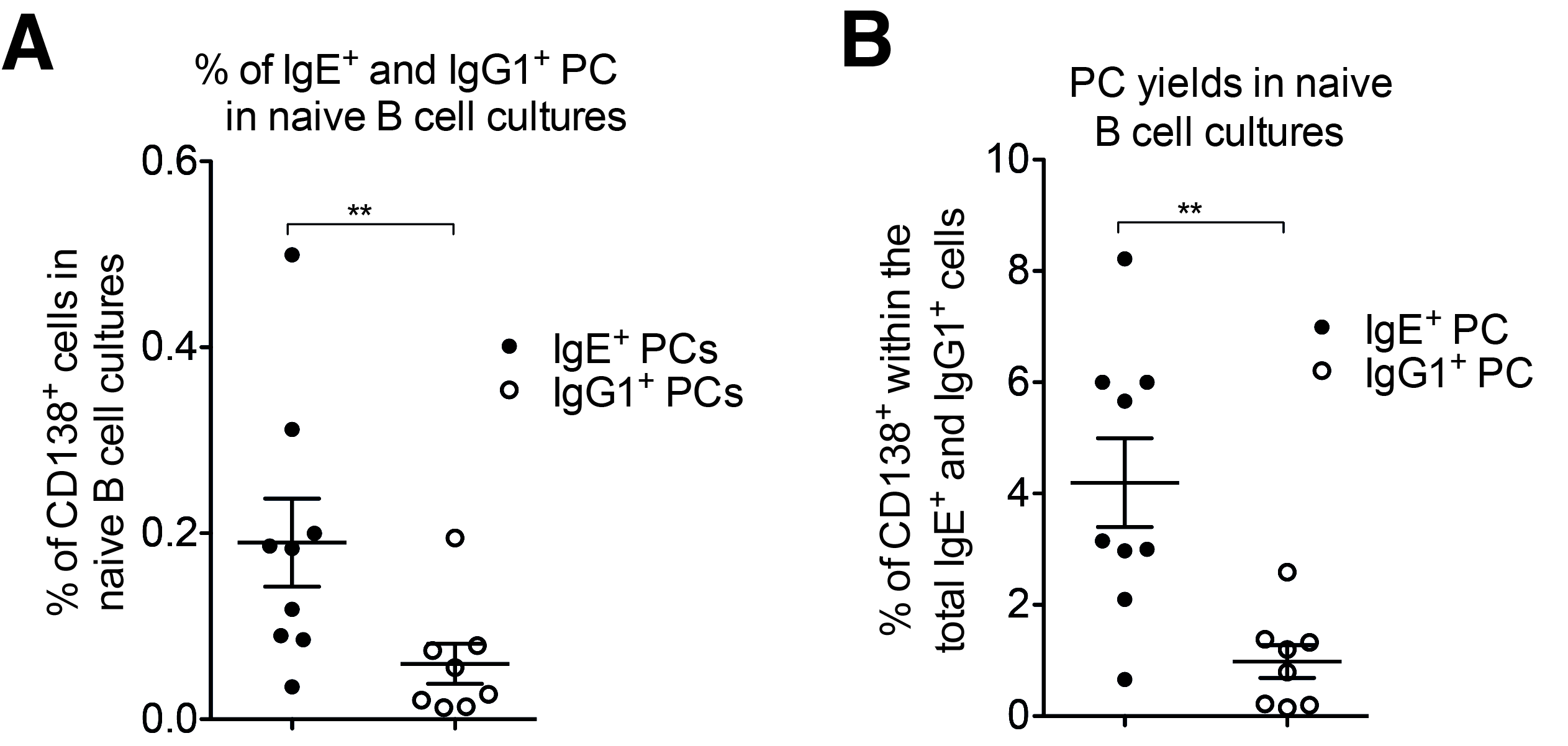


**Figure S5.** **Yields of IgE^+^ and IgG1^+^ PCs in naïve B cell cultures.** To determine the PC differentiation capacity of IgE^+^ and IgG1^+^ cells we stained cells, from day 10 of naïve B cell culture, for either IgE and CD138 or IgG1 and CD138. (**A**) The percentage of IgE^+^ PCs (IgE^+^CD138^+^) and IgG1^+^ PCs (IgG1^+^CD138^+^) in naïve B cell cultures. (**B**) Yields of IgE^+^ PCs (IgE^+^CD138^+^) and IgG1^+^ PCs (IgG1^+^CD138^+^) in naïve B cell cultures. The yields IgE^+^ PCs and IgG1^+^ PCs were calculated as a percentage of the total IgE^+^ and IgG1^+^ cells, respectively. Data represent the mean +/- SD. **p<0.01 (Two tailed t test).

**References**

1. Chen JB, Wu PC, Hung AF, et al. Unique epitopes on C epsilon mX in IgE-B cell receptors are potentially applicable for targeting IgE-committed B cells. *J Immunol*. 2010;184(4):1748-1756. Prepublished on 2010/01/20 as DOI 10.4049/jimmunol.0902437.

2. Ramadani F, Upton N, Hobson P, et al. Intrinsic properties of germinal center-derived B cells promote their enhanced class switching to IgE. *Allergy*. 2015. Prepublished on 2015/06/26 as DOI 10.1111/all.12679.

3. Iglesias-Ussel M, Marchionni L, Romerio F. Isolation of microarray-quality RNA from primary human cells after intracellular immunostaining and fluorescence-activated cell sorting. *J Immunol Methods*. 2013;391(1-2):22-30. Prepublished on 2013/02/26 as DOI 10.1016/j.jim.2013.02.003.
